# Association of communication methods and frequency with BMI among adolescents during the COVID-19 pandemic: findings from A-CHILD study

**DOI:** 10.3389/fpubh.2025.1433523

**Published:** 2025-02-28

**Authors:** Floret Maame Owusu, Nobutoshi Nawa, Hisaaki Nishimura, Yu Par Khin, Doi Satomi, Shiori Shakagori, Aya Isumi, Takeo Fujiwara

**Affiliations:** Department of Public Health, Institute of Science Tokyo, Tokyo, Japan

**Keywords:** adolescent health, BMI, communication style, COVID-19, Japan

## Abstract

**Objective:**

Little is known about the differential impact of communication methods and BMI. Hence, this study aims to examine the association of in-person and online communication with BMI among 13–14-year-old students during the COVID-19 pandemic.

**Method:**

This is a cross-sectional study which used data from the Adachi Child Health Impact of Living Difficulty study among Junior High School students in Adachi City, Tokyo in 2022(*N* = 3,178). A questionnaire was used to assess communication methods and frequency. BMI was categorized into overweight and obesity (≥ + 1SD), normal weight (−1SD to <+1SD) and underweight (<−1SD) based on WHO standard. Multinomial logistic regression was used to examine the association between communication methods and BMI.

**Results:**

Reduced in-person communication frequency was associated with 94% higher risk of overweight and obese (RRR = 1.94, 95%CI; 1.38, 2.72) while increased online communication frequency was associated with 46% increased risk (RRR = 1.46, 95%CI; 1.10, 1.95). When online and in-person communications were adjusted simultaneously, only reduced in-person communication frequency was associated with a high risk of overweight and obese (RRR = 1.56, 95%CI; 1.09, 2.25). When stratified by gender, a similar trend was observed among females (RRR = 2.12, 95%CI; 1.20, 3.73), but not in males.

**Conclusion:**

Reduced in-person communication frequency was associated with higher risk of overweight and obesity, especially among females, during COVID-19 in Japan.

## Introduction

The substantial increase in overweight and obesity prevalence among children and adults globally has become a public health issue of great concern. It is predicted that approximately 51% of people globally will be living with overweight or obesity by 2035 if action is not taken (1). Adolescent obesity has particularly been on the rise in recent times. As at 2022, 40 million adolescents and 39 million children worldwide were said to be obese (2). A systematic review on large cohorts showed that 80% of adolescents who are obese remain obese in adulthood (3). Unhealthy eating, lack of physical activity, stress and poor sleep quality which are risk factors for overweight and obesity were noticed to be aggravated during the COVID-19 pandemic, resulting in higher prevalence (4).

The COVID-19 pandemic, which induced school closures, lockdowns, movement restrictions and self-isolation, resulted in limited opportunity to engage in face-to face interactions. Adolescents resorted to using social media and other digital platforms to make up for the lack of social interactions and this led to a general increase in online communication (5). Tokyo prefecture in Japan, which is highly populated, had to close down schools earlier than most prefectures to control the spread of the COVID-19 (6). To allow continuity of learning despite the closure of schools, the government provided each child with personal computers (6). In 2021, the Tokyo metropolitan government even funded one-on-one online lessons in all public high schools in Tokyo (7). This new normal of online learning and digital interactions will encourage children and adolescents to spend more time on their phones and computers, resulting in a drastic reduction in face-to face interaction among their peers.

Face-to-face networks and interactions have been revealed to be advantageous in promoting healthy behaviors and lowering BMI (8). Also, literature has established that in the presence of their peers, children are influenced to engage more in physical activity (9). Face-to-face communication is beneficial in improving the health of adolescents, but in this era of alarming social media usage, social interactions may not be limited to face-to-face.

Over the years, digitalization has resulted in many adolescents turning to online platforms to interact. Existing research has suggested that online communication has helped adolescents build stronger peer relationships (10). Other studies done have however shown that excessive use of online platforms as means of communicating has led to poor health outcomes (11). A study done in China in 2017 confirmed that problematic smartphone use among primary and high schools students was positively associated with obesity (12). These are studies conducted in pre-pandemic era. There are however limited studies on its impact on BMI during the pandemic.

Interestingly, gender differences have been noticed to modify the effect online and in-person communications have on BMI. In 2015, a population-based study among adolescents in Canada suggested that heavy social media use was associated with higher BMI among males than females (13). A previous study done in Canada observed that reduced face-to-face social interaction was associated with abdominal and general obesity among women but lower risk of obesity among men (14). Furthermore, in this era where both online and in-person of communication are of great essence for daily interactions, it is important to comprehensively understand how both forms of communication are simultaneously associated with BMI. This will help in attaining a healthy balance with respect to the frequency of both forms of communication and provide a better picture of the effect of both communication types on the weight of children and adolescents. However, to the best of our knowledge, no study has been found to simultaneously examine the association of online and in-person communication. Therefore, the objective of this study is to examine the association between online and in-person communication on BMI among Junior High School students, and to stratify this association by gender.

## Materials and methods

### Sample

This current study is a cross-sectional study using data from the Adachi Child Health Impact of Living Difficulty (A-CHILD) study, which is representative of a regional population. The first wave of this study was started in 2015 as a complete-sample longitudinal study. It targeted first grade students in all 69 elementary schools in Adachi City in Tokyo and a follow up survey was conducted in 2016, 2018, 2020, and 2022 for children who participated in the first survey.

**Figure 1:**
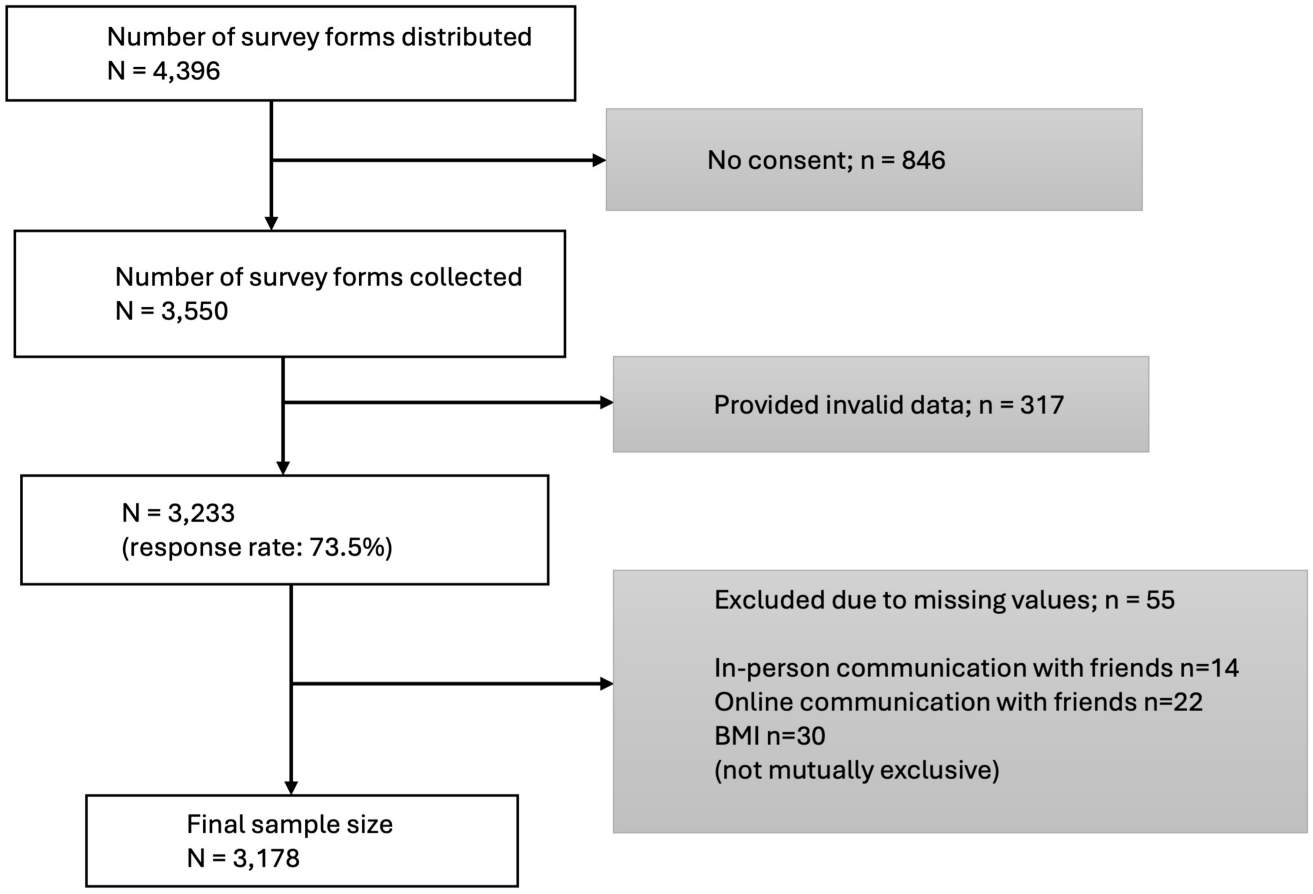
Flowchart.

In 2022, the follow up survey was conducted among second grade junior high school students (aged 13–14 years). For this survey, questionnaires were distributed among students and their caregivers (n = 4,396). The questionnaires, which were completed at home, were returned to school and submitted anonymously. Among the children who were given the questionnaires, 846 (19.2% of respondents) did not provide informed consent, thus these respondents were excluded from the study. After excluding responses with invalid data and missing values (*n* = 372), and linking with the school health checkup data, the final sample size used for the analysis was 3,178. The flowchart is shown in Figure 1.

This study was approved by the Ethics Committee at the National Center for Child Health and Development (Approval number: 1147) and Tokyo Medical and Dental University (Approval number: M2016-284).

### Measurements

#### Communication methods and frequency

The communication methods investigated were online and in-person communication. To examine the frequency of in-person communication, students were asked how often they had communicated with their friends in-person during the past month (excluding greetings). They were provided the following options as answers: 1 = every day, 2 = 4–6 days/week, 3 = 2–3 days/week, 4 = 0-1 day/week. For online communication, students were asked how often they had called their friends via online platforms over the past month. Similarly, they were provided the following options: 1 = every day, 2 = 4–6 days/week, 3 = 2–3 days/week, 4 = 0–1 day/week. In the final analysis, the categories 0–1 day/week and 2–3 days/week were combined to form a new category, 0–3 days/week for both online communication and in-person communication based on the distributions. This simplified categorization was designed to facilitate easier recall and reporting of communication frequencies among adolescent participants.

#### Body mass index (BMI)

The weight and height of the students were obtained in school by teachers during a mandatory school health checkup, following standardized protocol as outlined by the Japanese Society of School Health (15). Using a portable stadiometer, height was measured to the nearest 0.1 cm. Without shoes and in light clothing, weight was also measured to the nearest 0.1 kg on a digital scale. BMI, which was the outcome variable, was calculated by dividing the weight in kilograms (kg) by the square of the height in meters (m) and converted to z-score based on WHO standard (16). BMI was categorized into three groups as follows: overweight and obesity (≥ +1SD), normal weight (-1SD to < +1SD) and underweight (< -1SD).

#### Covariates

Information on the demographic characteristics of participants such as gender of students (male and female) and household income were obtained using self-reported questionnaires. Household income was categorized into <3,000,000 JPY, 3,000,000 JPY to 6,000,000 JPY, 6,000,000 JPY to10,000,000 JPY and > 10,000,000 JPY (1 USD ~ 132 JPY). The students were asked to report on the number of close friends they had inside of their class and outside of their class. For both questions, the response options were grouped into 0–4 friends, 5–9 friends and 10 or more friends based on distribution. This variable was made a covariate because social network size has been seen to be associated with physical activity and obesity (17), as well as communication (18). Previous studies have established that participation in sports club or extra-curricular activities is associated with physical fitness and BMI (19) and also influences communication competence (20). Involvement in athletic or sports club was therefore included as a covariate. Frequency of physical activity was also made a covariate. The Patient Health Questionnaire for Adolescents (PHQ-A) was used to evaluate depressive symptoms among students (21). Participants completed a 4-point Likert scale in response to 9 items with responses nearly every day, more than half the days, several days and not at all. The total PHQ-A score which could range from 0 to 27 were grouped into 0–4 for no or minimal depressive symptoms, 5–14 for mild to moderate depressive symptoms, 15–19 for moderate to severe depressive symptoms and 20–27 for severe depressive symptoms. Depression, which is associated with BMI (22) is also with communication (23) and so was made a covariate.

To assess the level of psychological distress among caregivers, the Japanese version of the K-6 scale was used (24). They were asked to answer a 6-item set of questions based on their experiences over the past 30 days using a 5-point Likert scale. The responses were summed up to obtain a score ranging from 0 to 24 and categorized as 0–4, 5–12 and 13–24, depicting low, moderate and high levels of psychological distress, respectively. Caregivers also had to report the absence or presence of maternal and paternal illness. Parental mental and physical health were made covariates because previous studies have established that the mental and physical health status of parents influence the BMI of their children (25). Literature has also shown that parental mental health impacts the mental health of children which subsequently affects communication skills (26). The physical health of parents has also been shown to influence communication skills among children (27). In addition, the order of birth of children was made a covariate due to previous research highlighting the association between birth order and BMI (28) and also birth order and communication (29). To assess their birth order, each child was asked whether they had an older sibling and a younger sibling. For both questions, the options given were ‘yes’ and ‘no’. The data from these responses were recoded and each child was categorized into only child (having no siblings), firstborn, middle born and last born. Finally, caregivers were asked if their children had been admitted at the hospital over the past 1 year. They answered, ‘yes’ or ‘no’. They were also asked if they had been absent from school since they entered their current grade with answer options ‘yes’, ‘no’ and ‘I do not know’. According to previous studies, BMI of children is associated with chronic illnesses (30) which in turn affects school attendance. School absenteeism has also been found to affect communication among peers (31). Hence, the covariates absence from school and previous hospital admissions were included.

### Statistical analysis

Multinomial logistic regression analysis was used to examine the association of online communication and in-person communication with BMI, using the reference categories ‘every day’ for in-person communication and ‘0–3 days/week’ for online communication. The covariates (gender of children, household income, close friends inside and outside the classroom, membership in athletics or sports club, frequency of exercise, PHQ-A score of children, children’s birth order, K-6 score of caregivers, maternal and paternal illness, history of hospital admission and absence from school) were included in the adjusted model. Lastly, the association was further stratified by the children’s gender (male and female) and was also adjusted for using all other covariates. All analyses were performed using the computer software STATA 16 for MacOS (STATA Corp., College Station, TX, USA).

## Results

Table 1 shows the characteristics of the caregivers and children. Among the three BMI categories, children who were overweight and obese were less likely to communicate in-person with their friends, (*n* = 54, 10.1%), less likely to be members of athletics and sports clubs, (*n* = 234, 43.8%), more likely to be the only child of their parents, (*n* = 133, 24.9%) and more likely to be male, (*n* = 323, 60.5%). Also, children from households with low income were more likely to be obese, (*n* = 85, 15.9%).

**Table 1:** Demographic characteristics of participants (N = 3,178).

		BMI Category		*p*-value
Demographic characteristics	Underweight (*N* = 514, 16.2%)	Normal weight (*N* = 2,130, 67.0%)	Overweight and obesity (*N* = 534, 16.8%)	
In-person communication with friends				
Everyday	346 (67.3%)	1,496 (70.2%)	342 (64.0%)	<0.001*
4–6 days/week	144 (28.0%)	512 (24.0%)	138 (25.8%)	
0–3 days/week	24 (4.7%)	122 (5.7%)	54 (10.1%)	
Online communication with friends				
Everyday	39(7.6%)	172(8.1%)	44 (8.2%)	0.12
4–6 days/week	60(11.7%)	209(9.8%)	73 (13.7%)	
0–3 days/week	415(80.7%)	1,749(82.1%)	417(78.1%)	
Membership in athletic and sports club				
Yes	323(62.8%)	1,409 (66.2%)	298 (55.8%)	<0.001*
No	186(36.2%)	705 (33.1%)	234 (43.8%)	
Missing	5(1.0%)	16 (0.8%)	2 (0.4%)	
Frequency of exercise				
Rarely	186(36.2%)	693 (32.5%)	191 (35.8%)	0.16
1–2 times/week	149(29.0%)	597 (28.0%)	164 (30.7%)	
3–4 times/week	82(16.0%)	373 (17.5%)	89 (16.7%)	
5–6 times/week	51(9.9%)	264 (12.4%)	49 (9.2%)	
Everyday	41(8.0%)	193 (9.1%)	40 (7.5%)	
Missing	5(1.0)	10 (0.5%)	1 (0.2%)	
Close friends in the same class				
0–4	167 (32.5%)	753 (35.4%)	180 (33.7%)	0.06
5–9	200 (38.9%)	719 (33.8%)	163 (30.5%)	
10+	141 (27.4%)	638 (30.0%)	186 (34.8%)	
Missing	6 (1.2%)	20 (0.9%)	5 (0.9%)	
Close friends outside the same class				
0–4	167 (32.5%)	697 (32.7%)	188 (35.2%)	0.56
5–9	183 (35.6%)	692 (32.5%)	173 (32.4%)	
10+	158 (30.7%)	716 (33.6%)	164 (30.7%)	
Missing	6 (1.2%)	25 (1.2%)	9 (1.7%)	
Absence from school				
Yes	283 (55.1%)	1,128 (53.0%)	320 (59.9%)	0.19
No	225 (43.8%)	979 (46.0%)	208 (39.0%)	
I do not know	5 (1.0%)	20 (0.9%)	5 (0.9%)	
Missing	1 (0.2%)	3 (0.1%)	1 (0.2%)	
Hospital admission				
Yes	20 (3.9%)	148 (7.0%)	41 (7.7%)	0.07
No	493 (95.9%)	1,977 (92.8%)	493 (92.3%)	
Missing	1 (0.2%)	5 (0.2%)	0 (0.0%)	
PHQ-A Score				
0–4	290 (56.4%)	1,029 (48.3%)	256 (47.9%)	0.03
5–14	196 (38.1%)	899 (42.2%)	227 (42.5%)	
15–19	18 (3.5%)	120 (5.6%)	33 (6.2%)	
20+	9 (1.8%)	79 (3.7%)	18 (3.4%)	
Missing	1 (0.2%)	3 (0.1%)	0 (0.0%)	
Birth order				
Only child	84 (16.3%)	380 (17.8%)	133 (24.9%)	0.01*
Firstborn	182 (35.4%)	706 (33.2%)	156 (29.2%)	
Middle born	68 (13.2%)	294 (13.8%)	61 (11.4%)	
Last born	180 (35.0%)	750 (35.2%)	184 (34.5%)	
Gender				
Male	309 (60.1%)	995 (46.7%)	323 (60.5%)	<0.001*
Female	205 (39.9%)	1,135 (53.3%)	211 (39.5%)	
Maternal illness				
Absent	144 (28.0%)	575 (27.0%)	150 (28.1%)	0.82
Present	370 (72.0%)	1,555 (73.0%)	384 (71.9%)	
Paternal illness				
Absent	159 (30.9%)	647 (30.4%)	153 (28.7%)	0.57
Present	332 (64.6%)	1,358 (63.8%)	355 (66.5%)	
Missing	23 (4.5%)	125 (5.9%)	26 (4.9%)	
Caregiver’s K6 score				
0–4	322 (62.7%)	1,353 (63.5%)	316 (59.2%)	0.13
5–12	148 (28.8%)	608 (28.5%)	171 (32.0)	
13–24	35 (6.8%)	119 (5.6%)	41 (7.7%)	
Missing	9 (1.8%)	50 (2.4%)	6 (1.1%)	
Income				
<3,000,000 JPY	48 (9.3%)	224 (10.5%)	85 (15.9%)	0.001*
3,000,000-6,000,000 JPY	131 (25.5%)	556 (26.1%)	175 (32.8%)	
6,000,000 - 10,000,000 JPY	195 (37.9%)	734 (34.5%)	157 (29.4%)	
>10,000,000 JPY	58 (11.3%)	283 (13.3%)	41 (7.7%)	
Missing	82 (16.0%)	333 (15.6%)	76 (14.2%)	

^*^indicates *p*-value < 0.05.

Table 2 shows the association between online and in-person communication. There was inconsistent association between online communication and in-person communication, for example, online communication for every day had higher percentage for those who communicated in-person every day (9.6%) and 0-3 days/week (7.0%), while 4–6 days/week group showed lower percentage (3.9%). The Spearman correlation analysis produced a Spearman’s rho of 0.076, which suggested only a weak association between both forms of communication.

**Table 2:** Association between online communication and in-person communication (*n* = 3,178).

Online communication frequency	In-person communication frequency			
	0–3 days/week (%)	4–6 days/week (%)	Everyday (%)	Total
0–3 days/week	168 (84.0)	683 (86.0)	1,730 (79.2)	2,581 (81.2)
4–6 days/week	18 (9.0)	80 (10.1)	244 (11.2)	342 (10.2)
Everyday	14 (7.0)	31 (3.9)	210 (9.6)	255 (8.0)
Total	200 (100)	794 (100)	2,184 (100)	3,178
Spearman’s rho				0.076*
Pearson’s Chi-squared				28.74 (*p* < 0.001)

*Correlation coefficient < 0.4 indicates weak correlation.

Table 3 shows the association between online and in-person communication with BMI using multinomial logistic regression analysis. In model 1, result of the association between online communication and BMI is shown. Compared to 0–3 days/week, those who communicated online 4–6 days/week had a 46% higher risk (RRR = 1.46, 95%CI; 1.10, 1.95) of being overweight and obese. There was no significant association between online communication and being underweight. Model 2 shows the association between in-person communication and BMI. Compared to communicating in-person every day, those who communicated 0–3 days/week had a 94% increased risk (RRR = 1.94, 95%CI; 1.38, 2.72) of being overweight and obese. When online communication and in-person communication were adjusted simultaneously in model 3, in-person communication of 0–3 days/week was also associated with a 56% increased risk (RRR = 1.56, 95%CI; 1.09, 2.25) of being overweight and obese but online communication was not associated. Further, the analysis was stratified by gender, based on the marginal interaction effect (p for interaction =0.06).

**Table 3:** Association between online communication, in person communication and BMI (*n* = 3,178).

		*n* (%)	Model 1	Model 2	Model 3
			RRR (95%CI)	RRR (95%CI)	RRR (95% CI)
Overweight and obesity (≥ +1SD) (*n* = 534)	Online				
	Everyday	44 (8.2)	1.07 (0.76, 1.52)		0.93 (0.65, 1.34)
	4–6 days/week	73 (13.7)	1.46 (1.10, 1.95)*		1.27 (0.94, 1.71)
	0–3 days/week	417 (78.1)	ref		ref
	In person				
	Everyday	342 (64.0)		ref	ref
	4–6 days/week	138 (25.8)		1.18 (0.94, 1.47)	1.11 (0.88, 1.40)
	0–3 days/week	54 (10.1)		1.94 (1.38, 2.72)*	1.56 (1.09, 2.25)*
	Membership in athletic and sports club				
	Yes	298 (55.8)			ref
	No	234 (43.8)			1.51 (1.21, 1.89)*
	Frequency of exercise				
	Rarely	191(35.8)			ref
	1–2 times/week	164 (30.7)			1.13 (0.88, 1.46)
	3–4 times/week	89 (16.7)			0.97 (0.71, 1.32)
	5–6 times/week	49 (9.2)			0.75 (0.52, 1.10)
	Everyday	40 (7.5)			0.89 (0.59, 1.34)
	Close friends in the same class				
	0–4	180 (33.7)			ref
	5–9	163 (30.5)			1.10 (0.84, 1.43)
	10+	186 (34.8)			1.55 (1.14, 2.12)*
	Close friends outside the same class				
	0–4	188 (35.2)			ref
	5–9	173 (32.4)			0.827 (0.66, 1.13)
	10+	164 (30.7)			0.65 (0.47, 0.90)*
	Absence from school				
	Yes	320 (59.9)			ref
	No	208 (39.0)			0.80 (0.65, 0.98)*
	I do not know	5 (0.9)			0.96 (0.35, 2.66)
	Hospital admission				
	Yes	41 (7.7)			ref
	No	493 (92.3)			0.896 (0.59, 1.25)
	PHQ-A Score				
	0–4	256 (47.9)			ref
	5–14	227 (42.5)			0.97 (0.78, 1.19)
	15–19	33 (6.2)			1.00 (0.65, 1.54)
	20+	18 (3.4)			0.83(0.48, 1.45)
	Birth order				
	Only child	133 (24.9)			ref
	Firstborn	156 (29.2)			0.68 (0.52, 0.88)*
	Middle born	61 (11.4)			0.64 (0.45, 0.91)*
	Last born	184 (34.5)			0.80 (0.61, 1.04)
	Gender				
	Male	323 (60.5)			ref
	Female	211(39.5)			0.52 (0.42, 0.64)*
	Maternal illness				
	Absent	150 (28.1)			ref
	Present	384 (71.9)			0.83 (0.64, 1.06)
	Paternal illness				
	Absent	153 (28.7)			ref
	Present	355 (66.5)			1.29 (1.00, 1.66)*
	Caregiver’s K6 score				
	0–4	316 (59.2)			ref
	5–12	171 (32.0)			1.10 (0.88, 1.36)
	13–24	41 (7.7)			1.16 (0.78, 1.72)
	Income				
	<3,000,000 JPY	85 (15.9)			ref
	3,000,000-6,000,000 JPY	175 (32.8)			0.82 (0.60, 1.13)
	6,000,000 - 10,000,000 JPY	157 (29.4)			0.61 (0.44, 0.84)*
	>10,000,000 JPY	41 (7.7)			0.43 (0.28, 0.66)*
Underweight (< -1SD) (*n* = 514)	Online				
	Everyday	39 (7.6)	0.96 (0.66, 1.37)		0.85 (0.58, 1.25)
	4–6 days/week	60 (11.7)	1.21 (0.89, 1.64)		1.10 (0.80, 1.51)
	0–3 days/week	415 (80.7)	ref		ref
	In person				
	Everyday	346 (67.3)		ref	ref
	4–6 days/week	144 (28.0)		1.22 (0.98, 1.51)	1.13 (0.90, 1.41)
	0–3 days/week	24 (4.7)		0.85 (0.54, 1.34)	0.78 (0.49, 1.24)
	Membership in athletic and sports club				
	Yes	323 (62.8)			ref
	No	186 (36.2)			1.19(0.94, 1.49)
	Frequency of exercise				
	Rarely	186 (36.2)	ref	ref	ref
	1–2 times/week	149 (29.0)			0.89 (0.695, 1.14)
	3–4 times/week	82 (16.0)			0.74 (0.55, 1.01)
	5–6 times/week	51 (9.9)			0.63 (0.44, 0.90)*
	Everyday	41 (8.0)			0.69 (0.47, 1.03)
	Close friends in the same class				
	0–4	167 (32.5)	ref	ref	ref
	5–9	200 (38.9)			1.17 (0.91, 1.51)
	10+	141 (27.4)			0.92 (0.67, 1.27)
	Close friends outside the same class				
	0–4	167 (32.5)	ref	ref	ref
	5–9	183 (35.6)			1.01 (0.78, 1.31)
	10+	158 (30.7)			0.84 (0.61, 1.16)
	Absence from school				
	Yes	283 (55.1)	ref	ref	ref
	No	225 (43.8)			0.87 (0.71, 1.07)
	I do not know	5 (1.0)			0.98 (0.36, 2.68)
	Hospital admission				
	Yes	20 (3.9)			ref
	No	493 (95.9)			1.82 (1.12, 2.96)*
	PHQ-A Score				
	0–4	290 (56.4)			ref
	5–14	196 (38.1)			0.78 (0.63, 0.96)*
	15–19	18 (3.5)			0.53 (0.31, 0.89)*
	20+	9 (1.8)			0.42 (0.20, 0.85)*
	Birth order				
	Only child	84 (16.3)			ref
	Firstborn	182 (35.4)			1.14 (0.85, 1.53)
	Middle born	68 (13.2)			1.01 (0.71, 1.45)
	Last born	180 (35.0)			1.06 (0.79, 1.43)
	Gender				
	Male	309 (60.1)			ref
	Female	205 (39.9)			0.53 (0.42, 0.65)*
	Maternal illness				
	Absent	144 (28.0)			ref
	Present	370 (72.0)			0.98 (0.76, 1.26)
	Paternal illness				
	Absent	159 (30.9)			ref
	Present	332 (64.6)			1.00 (0.78, 1.29)
	Caregiver’s K6 score				
	0–4	322 (62.7)			ref
	5–12	148 (28.8)			1.02 (0.82, 1.29)
	13–24	35 (6.81)			1.25 (0.83, 1.89)
	Income				
	<3,000,000 JPY	48 (9.3)			ref
	3,000,000-6,000,000 JPY	131(25.5)			1.08 (0.74, 1.57)
	6,000,000 - 10,000,000 JPY	195 (37.9)			1.24 (0.86, 1.79)
	>10,000,000 JPY	58 (11.3)			0.98 (0.63, 1.51)

RRR, Relative risk ratio; CI, Confidence interval.

Model 1 – bivariate regression between online communication and BMI.

Model 2 – bivariate regression between in person communication and BMI.

Model 3 – adjusted for sex, income, communication inside and outside the class, order of birth, depression and involvement in club activities, hospital admissions, absence from school, mental health of caregiver, maternal and paternal disease, and physical activity.

*indicates *p*-value < 0.05.

Table 4 shows the association between online and in-person communication with BMI among females. In model 1, compared to online communication of 0-3 days/week, communicating 4–6 days/week was associated with 70% increased risk of overweight and obesity (RRR = 1.70, 95%CI; 1.02, 2.85). In model 2, compared to talking every day, females who communicated in-person 4–6 days/week had 42% increased risk (RRR = 1.42, 95%CI; 1.02, 1.97) of being overweight and obese and those who communicated 0–3 days/week had 157% increased risk (RRR = 2.57, 95%CI; 1.53, 4.31) of being overweight and obese. In model 3, when online communication and in-person communication were adjusted simultaneously, the association remained similar to that of model 2. That is, compared to talking every day, in-person communication of 4–6 days/week was associated with 43% higher risk (RRR = 1.43, 95%CI; 1.01, 2.03) while 0–3 days/week was associated with 112% higher risk (RRR = 2.12, 95%CI; 1.20, 3.73) of being overweight and obese.

**Table 4:** Association between online communication, in person communication and BMI among female students (n = 1,551).

		*n* (%)	Model 1	Model 2	Model 3
			RRR (95%CI)	RRR (95%CI)	RRR (95% CI)
Overweight and obesity (≥ +1SD) (*n* = 211)	Online				
	Everyday	11 (5.2)	1.30 (0.66, 2.56)		1.14 (0.55, 2.34)
	4–6 days/week	21 (10.0)	1.70 (1.02, 2.85)*		1.60 (0.93, 2.74)
	0–3 days/week	179 (84.3)	ref		ref
	In person				
	Everyday	125 (59.2)		ref	ref
	4–6 days/week	63 (30.0)		1.42 (1.02, 1.97)*	1.43 (1.01, 2.03)*
	0–3 days/week	23 (10.9)		2.57 (1.53, 4.31)*	2.12 (1.20, 3.73)*
	Membership in athletic and sports club				
	Yes	91 (43.1)			ref
	No	119 (56.4)			1.65 (1.17, 2.34)*
	Frequency of exercise				
	Rarely	92 (43.6)			ref
	1–2 times/week	68 (32.2)			1.09 (0.75, 1.58)
	3–4 times/week	27 (12.8)			0.98 (0.60, 1.62)
	5–6 times/week	14 (6.6)			1.06 (0.55, 2.04)
	Everyday	10 (4.7)			1.12 (0.52, 2.41)
	Close friends in the same class				
	0–4	95 (45.0)			ref
	5–9	58 (27.5)			1.09 (0.74, 1.61)
	10+	56 (26.5)			2.38 (1.48, 3.85)*
	Close friends outside the same class				
	0–4	101 (47.9)			ref
	5–9	71 (33.7)			0.85 (0.58, 1.24)
	10+	38 (18.0)			0.62 (0.37, 1.05)
	Absence from school				
	Yes	129 (61.1)			ref
	No	81 (38.4)			0.77 (0.56, 1.06)
	I do not know	1 (0.5)			1.31 (0.14, 12.53)
	Hospital admission				
	Yes	13 (6.2)			ref
	No	198 (93.8)			0.95 (0.50, 1.80)
	PHQ-A Score				
	0–4	84 (39.8)			ref
	5–14	93 (44.1)			0.85 (0.60, 1.18)
	15–19	22 (10.4)			1.26 (0.72, 2.20)
	20+	12 (5.7)			1.10 (0.53, 2.28)
	Birth order				
	Only child	53 (25.1)			ref
	Firstborn	61 (28.9)			0.69 (0.45, 1.06)
	Middle born	24 (11.4)			0.74 (0.42, 1.28)
	Last born	73 (34.6)			0.78 (0.52, 1.18)
	Maternal illness				
	Absent	57 (27.0)			ref
	Present	154 (73.0)			0.77 (0.52, 1.14)
	Paternal illness				
	Absent	54 (25.6)			ref
	Present	148 (70.1)			1.50 (1.01, 2.23)
	Caregiver’s K6 score				
	0–4	125 (59.2)			ref
	5–12	71 (33.7)			1.20 (0.85, 1.67)
	13–24	14 (6.6)			1.16 (0.61, 2.23)
	Income				
	<3,000,000 JPY	30 (14.2)			ref
	3,000,000-6,000,000 JPY	81 (38.4)			1.32 (0.80, 2.18)
	6,000,000 - 10,000,000 JPY	53 (25.1)			0.64 (0.38, 1.07)
	>10,000,000 JPY	17 (8.1)			0.58 (0.30, 1.15)
Underweight (< -1SD) (*n* = 205)	Online				
	Everyday	5 (2.4)	0.57 (0.22, 1.44)		0.58 (0.22, 1.54)
	4–6 days/week	13 (6.3)	1.01 (0.55, 1.86)		1.22 (0.65, 2.29)
	0–3 days/week	187 (91.2)	ref		ref
	In person				
	Everyday	149 (72.7)		ref	ref
	4–6 days/week	49 (23.9)		0.92 (0.65, 1.31)	0.87 (0.60, 1.25)
	0–3 days/week	7 (3.4)		0.65 (0.29, 1.46)	0.64 (0.28, 1.46)
	Membership in athletic and sports club				
	Yes	113 (55.1)			ref
	No	91(44.4)			1.08 (0.77, 1.52)
	Frequency of exercise				
	Rarely	92 (44.9)			ref
	1–2 times/week	63 (30.7)			0.86 (0.59, 1.25)
	3–4 times/week	32 (15.6)			0.93 (0.59, 1.49)
	5–6 times/week	8 (3.9)			0.42 (0.19, 0.92)*
	Everyday	10 (4.9)			0.79 (0.38, 1.67)
	Close friends in the same class				
	0–4	96 (46.8)			ref
	5–9	76 (37.1)			1.04 (0.73, 1.48)
	10+	30 (14.6)			0.80 (0.48, 1.35)
	Close friends outside the same class				
	0–4	88 (42.9)			ref
	5–9	80 (39.0)			1.14 (0.79, 1.63)
	10+	34 (16.6)			0.86 (0.51, 1.43)
	Absence from school				
	Yes	111 (54.2)			ref
	No	89 (43.4)			0.86 (0.63, 1.18)
	I do not know	4 (2.0)			5.43 (1.33, 22.17)*
	Hospital admission				
	Yes	9 (4.4)			ref
	No	196 (95.6)			1.31(0.63, 2.72)
	PHQ-A Score				
	0–4	103 (50.2)			ref
	5–14	88 (42.9)			0.72 (0.52, 0.99)*
	15–19	9 (4.4)			0.52 (0.25, 1.10)
	20+	5 (2.4)			0.55 (0.21, 1.45)
	Birth order				
	Only child	38 (18.5)			ref
	Firstborn	73 (35.6)			1.03 (0.66, 1.59)
	Middle born	27 (13.2)			1.05 (0.60, 1.83)
	Last born	67 (32.7)			0.82 (0.53, 1.29)
	Maternal illness				
	Absent	51 (24.9)			ref
	Present	154 (75.1)			1.27 (0.85, 1.90)
	Paternal illness				
	Absent	66 (32.2)			ref
	Present	128 (62.4)			0.77 (0.53, 1.13)
	Caregiver’s K6 score				
	0–4	141 (68.8)			ref
	5–12	49 (23.9)			0.84 (0.58, 1.20)
	13–24	12 (5.9)			1.26 (0.64, 2.49)
	Income				
	<3,000,000 JPY	15 (7.3)			ref
	3,000,000-6,000,000 JPY	60 (29.3)			1.84 (0.98, 3.45)
	6,000,000 - 10,000,000 JPY	82 (40.0)			1.66 (0.89, 3.07)
	>10,000,000 JPY	23 (11.2)			1.34 (0.65, 2.79)

RRR, Relative risk ratio; CI, Confidence interval.

Model 1 – bivariate regression between online communication and BMI.

Model 2 – bivariate regression between in person communication and BMI.

Model 3 – adjusted for sex, income, communication inside and outside the class, order of birth, depression and involvement in club activities, hospital admissions, absence from school, mental health of caregiver, maternal and paternal disease, and physical activity.

* indicates *p*-value < 0.05.

In Table 5, the results of multinomial logistic regression analysis to examine the association between online and in-person communication with BMI among males is shown. In model 1, there was no significant association between online communication and BMI. In model 2, in-person communication of 4–6 days/week was associated with a 48% higher risk (RRR = 1.48, 95%CI;1.11, 1.97) of being underweight. When online communication and in-person communication were adjusted simultaneously in model 3, there was no association between both communication methods and BMI.

**Table 5:** Association between online communication, in person communication and BMI among male students (n = 1,627).

		*n* (%)	Model 1	Model 2	Model 3
			RRR (95%CI)	RRR (95%CI)	RRR (95% CI)
Overweight and obesity (≥ +1SD) (*n* = 323)	Online				
	Everyday	33 (10.2)	0.82(0.54, 1.23)		0.80 (0.52, 1.23)
	4–6 days/week	52 (16.1)	1.15(0.81, 1.63)		1.16 (0.81, 1.67)
	0–3 days/week	238(73.7)	ref		ref
	In person				
	Everyday	217 (67.2)		ref	ref
	4–6 days/week	75 (23.2)		1.06 (0.78, 1.43)	0.92 (0.67, 1.26)
	0–3 days/week	31 (9.6)		1.54 (0.98, 2.43)	1.29 (0.79, 2.09)
	Membership in athletic and sports club				
	Ye	207 (64.1)			ref
	No	115 (35.6)			1.47 (1.08, 2.00)*
	Frequency of exercise				
	Rarely	99 (30.7)			ref
	1–2 times/week	96 (29.7)			1.12 (0.78, 1.60)
	3–4 times/week	62 (19.2)			0.93 (0.62, 1.38)
	5–6 times/week	35 (10.8)			0.64 (0.40, 1.02)
	Everyday	30 (9.3)			0.80 (0.48, 1.33)
	Close friends in the same class				
	0–4	85 (26.3)			ref
	5–9	105 (32.5)			1.08 (0.74, 1.59)
	10+	130 (40.3)			1.23 (0.80, 1.87)
	Close friends outside the same class				
	0–4	87 (26.9)			ref
	5–9	102 (31.6)			0.84 (0.57, 1.24)
	10+	126 (39.0)			0.65 (0.42, 1.01)
	Absence from school				
	Yes	191 (59.1)			ref
	No	127 (39.3)			0.80 (0.61, 1.05)
	I do not know	4 (1.2)			0.74 (0.23, 2.31)
	Hospital admission				
	Yes	28 (8.7)			ref
	No	295 (91.3)			0.84 (0.52, 1.34)
	PHQ-A Score				
	0–4	172 (53.3)			ref
	5–14	134 (41.5)			1.06 (0.81, 1.40)
	15–19	11 (3.4)			0.61 (0.30, 1.24)
	20+	6 (1.9)			0.49 (0.19, 1.23)
	Birth order				
	Only child	80 (24.8)			ref
	Firstborn	95 (29.4)			0.67 (0.47, 0.97)*
	Middle born	37 (11.5)			0.57 (0.36, 0.91)*
	Last born	111 (34.4)			0.82 (0.57, 1.17)
	Maternal illness				
	Absent	93 (28.8)			ref
	Present	230 (71.2)			0.83 (0.59, 1.16)
	Paternal illness				
	Absent	99 (30.7)			ref
	Present	207 (64.1)			1.17 (0.83, 1.64)
	Caregiver’s K6 score				
	0–4	191 (59.1)			ref
	5–12	100 (31.0)			1.03 (0.76, 1.38)
	13–24	27 (8.4)			1.21 (0.73, 2.01)
	Income				
	<3,000,000 JPY	55 (17.0)			ref
	3,000,000-6,000,000 JPY	94 (29.1)			0.55 (0.36, 0.84)*
	6,000,000 - 10,000,000 JPY	104 (32.2)			0.61 (0.40, 0.93)*
	>10,000,000 JPY	24 (7.4)			0.36 (0.20, 0.63)*
Underweight (< -1SD) (*n* = 309)	Online				
	Everyday	34 (11.0)	0.88 (0.59, 1.32)		0.91 (0.60, 1.39)
	4–6 days/week	47 (15.2)	1.09 (0.76, 1.56)		1.12 (0.77, 1.63)
	0–3 days/week	228 (73.8)	ref		ref
	In person				
	Everyday	197 (63.8)		ref	ref
	4–6 days/week	95 (30.7)		1.48 (1.11,1.97)*	1.31 (0.97, 1.78)
	0–3 days/week	17 (5.5)		0.93 (0.53, 1.62)	0.87 (0.48, 1.55)
	Membership in athletic and sports club				
	Yes	210 (68.0)			ref
	No	95 (30.7)			1.27 (0.92, 1.76)
	Frequency of exercise				
	Rarely	94 (30.4)			ref
	1–2 times/week	86 (27.8)			1.00 (0.69, 1.43)
	3–4 times/week	50 (16.2)			0.71 (0.47, 1.09)
	5–6 times/week	43 (13.9)			0.79 (0.50, 1.25)
	Everyday	31 (10.0)			0.75 (0.45, 1.25)
	Close friends in the same class				
	0–4	71 (23.0)			ref
	5–9	124 (40.1)			1.35 (0.92, 2.00)
	10+	111 (35.9)			1.05 (0.68, 1.63)
	Close friends outside the same class				
	0–4	79 (25.6)			ref
	5–9	103 (33.3)			0.85 (0.57, 1.26)
	10+	124 (40.1)			0.74 (0.48, 1.15)
	Absence from school				
	Yes	172 (55.7)			ref
	No	136 (44.0)			0.86 (0.66, 1.13)
	I do not know	1 (0.3)			0.20 (0.03, 1.56)
	Hospital admission				
	Yes	11 (3.6)			ref
	No	297 (96.1)			2.32 (1.20, 4.49)*
	PHQ-A Score				
	0–4	187 (60.5)			ref
	5–14	108 (35.0)			0.81 (0.61, 1.08)
	15–19	9 (2.9)			0.52 (0.24, 1.12)
	20+	4 (1.3)			0.32 (0.11, 0.93)
	Birth order				
	Only child	46 (14.9)			ref
	Firstborn	109 (35.3)			1.23 (0.82, 1.84)
	Middle born	41 (13.3)			0.97 (0.60, 1.58)
	Last born	113 (36.6)			1.28 (0.85, 1.91)
	Maternal illness				
	Absent	93 (30.1)			ref
	Present	216 (69.9)			0.79 (0.56, 1.11)
	Paternal illness				
	Absent	93 (30.1)			ref
	Present	204 (66.0)			1.25 (0.88, 1.77)
	Caregiver’s K6 score				
	0–4	181 (58.6)			ref
	5–12	99 (32.0)			1.20 (0.90, 1.62)
	13–24	23 (7.4)			1.36 (0.80, 2.31)
	Income				
	<3,000,000 JPY	33 (10.7)			ref
	3,000,000-6,000,000 JPY	71 (23.0)			0.69 (0.42, 1.12)
	6,000,000 - 10,000,000 JPY	113 (36.6)			1.01 (0.63, 1.62)
	>10,000,000 JPY	35 (11.3)			0.77 (0.43, 1.35)

RRR, Relative risk ratio; CI, Confidence interval.

Model 1 – bivariate regression between online communication and BMI.

Model 2 – bivariate regression between in person communication and BMI.

Model 3 – adjusted for sex, income, communication inside and outside the class, order of birth, depression and involvement in club activities, hospital admissions, absence from school, mental health of caregiver, maternal and paternal disease, and physical activity.

* indicates *p*-value < 0.05.

Further, analysis using interaction between communication methods and household income showed no significant association across all income categories.

## Discussion

In this study, we found that reduced frequency in in-person communication was associated with a higher risk of becoming overweight or obese during COVID-19 pandemic. We also found that female adolescents were at an increased risk of overweight and obesity with a reduction in the frequency of in-person communication while males were not. To the best of our knowledge, this is the first study to examine the effect of communication methods and frequency on the BMI of adolescents during the COVID-19 pandemic, and to simultaneously examine the association of online and in-person communication with BMI.

Our finding on reduced in-person communication frequency being associated with a higher risk in becoming overweight and obese among adolescents was not reported in other studies, since previous studies conducted on adolescent and young adults focused on social networks which combined both online and in-person communications (32, 33). Among peers, social facilitation plays an important role in influencing certain health behaviors, such as physical activities (34). A previous study revealed that adolescents are motivated to engage in physical activities in the presence of their friends (35). While being physically present with their peers and communicating face-to-face, adolescents exhibit more physical activities. A reversal of this situation could potentially lead to a decrease in face-to-face communication and consequently, a decrease in physical activity. Additionally, physical communication among peers could enhance sharing of knowledge and information that promote healthy behaviors. A previous study conducted in a manufacturing firm revealed that face-to-face social networks and interactions significantly promote sharing of knowledge among groups and organizations (36). Although the reference focuses on knowledge sharing in companies, the underlying principles of face-to-face interactions facilitating information sharing and influencing behavior could be applicable to in-person interactions in school settings. These interactions are important for disseminating health-related knowledge that contribute to maintaining a healthy BMI.

From our study, female adolescents were at an increased risk of overweight and obesity with a reduction in in-person communication frequency while males were not. A similar study conducted among adults aged 45–85 years demonstrated that reduced social participation was associated with weight gain among females but not in males (14), which confirms our finding. To the best of our knowledge, there was however no study found specific to adolescents. Our finding among adolescents could be explained in several ways. First, it is well established that adolescent females generally have higher body fat levels than males and are therefore more prone to weight gain (37). Second, concerning extracurricular activities, females are less likely to participate in afterschool physical activities compared to males (38). Instead, they are inclined to join groups that involve minimal physical energy such as academic or arts groups (39). Reduction in in-person interaction further decreases their physical activity levels, leading to an increase in BMI. Third, in Japan, females’ perception of their body image and desire to be thin are influenced by their peers (40). While meeting and interacting with their peers in school, they tend to be more self-aware of their body size. Consequently, a decrease in peer interaction might lead to a reduced concern about their body image, possibly resulting in weight gain.

From our findings, we observed inconsistent results between the adjusted and crude models for online communication and BMI. In the crude model, we observed an association between online communication and BMI, which we acknowledge was biased by confounders. To account for this, we adjusted for confounders. When the adjusted model did not show any significant result, we interpreted this as indicating that the association observed in the crude model was confounded by these variables. The true association is observed in the adjusted model, revealing no association between online communication and BMI.

Our study has some limitations; first, the frequency of online and in-person communication was self-reported by participants, and assessed retrospectively which could result in non-differential misclassification of the exposure and recall bias. To mitigate this, future studies could incorporate cross-validation with alternative data collection methods such as digital communication logs, wearable technology or attendance records such as club participation logs to infer face-to-face interaction. Second, this study exclusively involved children from public schools in Adachi city, which may limit the generalizability of our findings. In Japan, children attending private schools are more often from higher socio-economic backgrounds compared to those in public schools (41). This socio-economic difference may provide students from private schools with better access to technology and extracurricular activities, which could influence their communication methods and health outcomes. However, the proportion of students in private elementary school is very low, 1.1% (42). In addition, since Adachi City is predominantly urban, public schools in rural areas may differ from urban areas in terms of access to technology for communication and classroom sizes. These could also affect communication methods. Future studies could include a more diverse range of schools, encompassing both public and private high schools in both rural and urban settings. Lastly, because this is a cross-sectional study, we cannot establish causality between in-person communication and overweight/obesity, implying the possibility of reverse causation. For instance, children with high BMI are less likely to engage in face-to-face communication with their peers (43), rather than reduced in-person communication resulting in higher BMI. To address this limitation, future studies using longitudinal design should be conducted to understand the causal pathway of this association. Despite these limitations, findings from this study can serve as a valuable resource for public health policy makers in shaping policies aimed at enhancing the physical well-being of children in the post covid-19 era and in future pandemics. This could be done by setting up initiatives to promote physical interactions within schools and communities. Additionally, it can offer guidance to parents and teachers on promoting healthy lifestyle by encouraging increased face-to-face interactions in school and at home as opposed to online social networking. Further, findings from this study could be used in health promotion campaigns to help children understand the importance of physically engaging with friends in school.

## Conclusion

Our study revealed that reduced frequency in face-to face interaction was associated with overweight and obesity, particularly among females, during the COVID-19 pandemic. These findings may offer valuable insights into the importance for face-to-face interactions in the post-COVID-19 era and future pandemics, aiming to tackle potential health concerns among children and adolescents.

## Data Availability

The datasets presented in this article are not publicly available due to ethical restrictions. Requests to access the datasets should be directed to fujiwara.hlth@tmd.ac.jp.
